# The research progress of Chikungunya fever

**DOI:** 10.3389/fpubh.2022.1095549

**Published:** 2023-01-09

**Authors:** Li Cai, Xinyi Hu, Shuang Liu, Lei Wang, Hao Lu, Hua Tu, Xibao Huang, Yeqing Tong

**Affiliations:** ^1^Department of Infectious Disease Control and Prevention, Wuhan Center for Disease Control and Prevention, Wuhan, China; ^2^School of Public Health, Wuhan University, Wuhan, China; ^3^Global Study Institute, University of Geneva, Geneva, Switzerland; ^4^Department of Infectious Disease Control and Prevention, Hubei Center for Disease Control and Prevention, Wuhan, China; ^5^Department of Economic Management, China University of Geosciences, Wuhan, China

**Keywords:** Chikungunya, pathogenesis, vaccine, epidemiology, prevention

## Abstract

Chikungunya fever, an acute infectious disease caused by Chikungunya virus (CHIKV), is transmitted by Aedes aegypti mosquitoes, with fever, rash, and joint pain as the main features. 1952, the first outbreak of Chikungunya fever was in Tanzania, Africa, and the virus was isolated in 1953. The epidemic has expanded from Africa to South Asia, the Indian Ocean islands and the Americas, and is now present in more than 100 countries and territories worldwide, causing approximately 1 million infections worldwide each year. In addition, fatal cases have been reported, making CHIKV a relevant public health disease. The evolution of the virus, globalization, and climate change may have contributed to the spread of CHIKV. 2005–2006 saw the most severe outbreak on Reunion Island, affecting nearly 35% of the population. Since 2005, cases of Chikungunya fever have spread mainly in tropical and subtropical regions, eventually reaching the Americas through the Caribbean island. Today, CHIKV is widely spread worldwide and is a global public health problem. In addition, the lack of a preventive vaccine and approved antiviral treatment makes CHIKV a major global health threat. In this review, we discuss the current knowledge on the pathogenesis of CHIKV, focusing on the atypical disease manifestations. We also provide an updated review of the current development of CHIKV vaccines. Overall, these aspects represent some of the most recent advances in our understanding of CHIKV pathogenesis and also provide important insights into the current development of CHIKV and potential CHIKV vaccines for current development and clinical trials.

## 1. Introduction

Chikungunya virus (CHIKV) is a single positive-standard RNA virus in the alphavirus genus of the family of Togaviridae (1). The genome is about 11.8kb in length, including the 5′ -terminal non-coding region (5′ UTR), two independent open reading frames (ORF), 1 poly A tail and 3′ UTR, encoding four non-structural proteins (nsP1, nsP2, nsP3, nsP4) and six structural proteins (capsid protein C, envelope protein E3, E2,6K, E1, and TF). The genes encoding the viral envelope proteins have been mutated in recent decades, which facilitated CHIKV transmission by Aedes albopictus (2). CHIKV has four genotypes: East-Central-South African genotype (ECSA), West African genotype, Asian genotype and Indian OceanLineage genotype (IOL). Among them, the Asian and Indian Ocean Lineage genotypes were derived from ECSA. The ECSA and Asian genotypes are mainly transmitted by Aedes aegypti, and the Indian Ocean genotype has acquired the ability (3, 4) to transmit through Aedes albopictus after genetic mutations occur. CHIKV can be inactivated by 70% ethanol, l% sodium hypochlorite, 2% glutaraldehyde, and lipid solvents, peracetic acid, and other disinfectants. CHIKV is relatively stable at minus 40 degrees, and it can be inert by heating to above 58 degrees. Although CHIKV infection is usually a self-limiting disease, some patients develop persistent joint pain after the acute phase that may last for months or years (5, 6).

## 2. Epidemiology

### 2.1. Source of infection

In the urban epidemic source sites, patients and hidden infected persons are the main sources of infection, and their transmission mode is mainly human-mosquito-human. In the early stage of the disease, 2–5 d can produce a high titer of viremia, which is very infectious. In the jungle epidemic foci, infected monkeys, orangutans, baboons and other primates, and wild animals are the main sources of infection of the disease, and the mode of transmission is mainly primate-mosquito-primate (7).

### 2.2. Route of transmission

Aedes, including Aedes albopictus mosquitoes, Aedes aegypti mosquitos, and African Aedes mosquitoes, are the primary vectors of CHIKV, Aedes albopictus originated in Asia and is a wild mosquito species distributed in tropical, subtropical, and temperate rural and urban areas, and is currently distributed globally. Its eggs are drought tolerant and, therefore resilient, and have surpassed Aedes aegypti as the main local mosquito species in many areas. Aedes aegypti is a domestic mosquito species widely distributed in tropical and subtropical cities and suburbs. It mainly breeds from the water in small containers in residential areas. Aedes africanus is an African wild mosquito species, addicted to primate blood and spreads viruses in wild animals, playing a major role in virus circulation in jungle-type foci (8, 9).

### 2.3. Susceptibility and immunity

The population is generally susceptible to CHIKV and can develop at any age. After infection, dominant infection and recessive infection, the latter majority, and both can obtain immunity.

### 2.4. Epidemiological characteristics

#### 2.4.1. Regional distribution

Since the first CHIKV infection case was reported in Africa in the 1950s, subsequent epidemics mainly outbreak in the sub-Saharan region of Africa, Southeast Asia, South Asia, the Indian Ocean islands, and the Western Pacific region. In 2007, the first European case was reported in Italy. In late 2013, the first local transmission case was confirmed in the Americas, demonstrating that mosquitos in these areas also got infected with CHIKV and spread to humans. In 2022, over 250 thousand autochthonous suspected and confirmed cases have been reported in the Americas. The cross-regional prevalence of epidemics indicated vectors adapted to more temperate regions in Europe and America.

#### 2.4.2. Population distribution

The population distribution of Chinkungunya infection for native and imported cases is different. All genders and age groups are highly susceptible to infection before Chikungunya fever becomes an endemic disease. When indigenous transmission occurs, patients at the extreme age spectrum are to some extent more susceptible and at higher risk to developed severe symptoms.

Seasonal prevalence is consistent with the breeding season of the vector mosquitoes. Popular seasons mainly concentrated on the rainy season with high temperature and high humidity. In subtropical and temperate regions, summer and autumn are the popular seasons. In Asia, the first case of CHIKV was reported in Cambodia in 1961, probably with (10) caused by an Asian genotype circulating in the region at the time. Forty years later, Chikungunya fever outbreak again in Sri Lanka in 2007, causing more than 37,000 infected cases (11). The intensification and expansion of vector-borne diseases may be a major threat to climate change. In fact, despite many other complex factors (such as mosquito-range constraints and virus evolution), climate change will cause increased (12, 13) exposure to Aedes-borne viruses.

## 3. Pathogenesis

CHIKV belongs to the “New World” group of alphaviruses, which mainly cause musculoskeletal inflammatory diseases such as arthralgia, arthritis, and myalgia, classified as “arthritis virus”, including Chikunonia, Ross River virus (RRV), Ballmach Forest virus (BFV), Group Sindbis and Mayaro (Mayv) (14, 15). CHIKV infects multiple cell types, including dendritic cells, and macrophages. Synovial fibroblasts, endothelial cells, and myocytes. In the human body, osteoblasts can also be infected, causing arthropathy and erosive disease (16) in patients with chronic arthritis.

On the viral surface, the heterodi-and trimers of the structural proteins E1 and E2 proteins form “viral spikes”, and the glycoprotein E2 is responsible for binding to the receptor, while E1 is responsible for the fusion of (17) to the membrane. Given that CHIKV infects multiple types of cells, the cellular proteins that interact with the virus are also diverse in (18). Mammalian cell receptors known for CHIKV include prohibition (PHB), TIM-1, MXRA8, CD147, lectin DC-SIGN, and ATP synthase subunit and FHL1 (19–25). Furthermore, other phosphatidylserine-binding proteins, such as Axl, TIM-4 and TIM-1, have also been shown to promote (26–30) in CHIKV-infected cells. Otherwise, autophagy apoptosis is an important infection mechanism. It has been shown that CHIKV initiates apoptosis through both endophytic and exogenous pathways in HeLa cells, as well as in primary fibroblasts. By hiding within these apoptotic bleeding sites, CHIKV is able to infect adjacent cells. These infectious foci infect macrophages like Trojan viruses, and interestingly, the replication process of this cell-infected virus in macrophages does not produce a proinflammatory response, constituting the mechanism (31), by which CHIKV invades the host cell and escapes the host response. After infection, the incubation period is 3–7 days, in the acute phase, and the most common symptoms are high fever, stiffness, headache, photophobia and ecchymosis rash or punctate rash. Similar to other arboviruses, DENV and ZIKV, the peak of viremia in CHIKV-infected people matched the duration of fever for (32–34), and hospitalized cases had higher viremia than those that did not require hospitalization. Roughly estimated, 30–40% of infected individuals experience some long-term sequelae, including persistent arthralgia and / or arthritis, with about 37% having severe persistent arthralgia. Some studies show that patients older than 40 years, of the female gender, with higher levels of CXCL8, detected in the acute phase of the disease have been shown to associate (35–37) with persistent arthralgia in CHIK. CHIKV susceptibility and *in vitro* single-cell correlation studies showed that the arthritis-related genes RANTES / CCL5 and IL-8 significantly upregulate (38) in infected human synovial fibroblasts. Following viral replication *in vivo*, the cells target muscle, joints, and skin fibroblasts, and these tissue cell damage was also observed in human biopsy samples. A study of neonatal and adult mouse models with IFN knockout found that the severity of CHIKV infection was (39) associated with the IFN- / R signaling pathway.

## 4. Vaccines and treatment

Despite the prevalence of CHIKV in many regions, no marketed vaccine is available. Treatment of CHIKV-infected patients mainly provides symptom relief through the use of anti-inflammatory drugs, such as methotrexate, sulfasalazine, leflunomide, and hydroxychloroquine, which has been utilized but with limited efficacy (40, 41). A wide range of CHIKV vaccine candidates under preclinical development, including whole-virus inactivated vaccine (42, 43), VEE / CHIKV chimeric vaccine, recombinant adenovirus vector vaccine, DNA-and mRNA-based CHIKV vaccine, and the live attenuated vaccine (44–50) with stronger and longer immune response, are currently in clinical research phase I-III, and we need to focus on the antibody dependence (ADE) phenomenon (51), with a breakthrough in future vaccine development.

## 5. Outlook

CHIKV is spreading rapidly in many countries, and the vaccination of susceptible people is the most effective way to control the infection, and we can expect a CHIKV vaccine to be available to the general public in perhaps 5–10 years. Before vaccines and specific drugs are put on sale, we still need to strengthen CHIKV testing for suspected acute neurological symptoms and conduct timely detection of cases and treatment of patients to contain the spread of the epidemic.

## Author contributions

YT, LC, and XinH: conceptualization. XibH and XinH: methodology. HL and HT: resources. YT and XinH: writing-original draft preparation. LC: writing-review and editing. LW: visualization. XibH: supervision. SL: project administration. YT: funding acquisition. All authors contributed to the article and approved the submitted version.
